# Association of uric acid levels with severity of *Plasmodium* infections: a systematic review and meta-analysis

**DOI:** 10.1038/s41598-023-42217-8

**Published:** 2023-09-11

**Authors:** Saruda Kuraeiad, Kwuntida Uthaisar Kotepui, Frederick Ramirez Masangkay, Aongart Mahittikorn, Manas Kotepui

**Affiliations:** 1 Medical Technology, School of Allied Health Sciences, Walailak University, Tha Sala, Nakhon Si Thammarat, Thailand; 2 Research Center in Tropical Pathobiology, Walailak University, Nakhon Si Thammarat, Thailand; 3 Department of Medical Technology, Faculty of Pharmacy, University of Santo Tomas, Manila, Philippines; 4 Department of Protozoology, Faculty of Tropical Medicine, Mahidol University, Bangkok, Thailand

**Keywords:** Diagnostic markers, Prognostic markers, Malaria

## Abstract

Elevated uric acid (UA) levels have been reported in malaria patients and are particularly prominent in severe malaria cases. This study aims to synthesize the difference in UA levels between malaria patients and uninfected controls, and between patients with severe and non-severe malaria. A comprehensive literature search was carried out across databases such as Embase, MEDLINE, Ovid, PubMed, Scopus, ProQuest, and Google Scholar to identify relevant studies for inclusion. The methodological quality of the included studies was evaluated independently by two reviewers using the JBI critical appraisal tool for observational studies. A meta-analysis was performed to calculate the pooled effect sizes, expressed as Hedges' g, with 95% confidence intervals (CIs). The Hedges' g was pooled using the random-effects model. An initial search yielding a total of 1122 articles, and a final total of 19 studies being included in the review. Elevated UA levels were observed more prominently in malaria patients, especially those with severe manifestations, when compared to uninfected controls. The conducted meta-analysis demonstrated a significant elevation in UA levels in patients suffering from malaria as compared to uninfected controls (*P* < 0.01, Hedges’s g = 1.40, 95% CI 0.84–1.95, *I*^2^ = 95.81, 16 studies). The conducted meta-analysis demonstrated a significant elevation in UA levels in patients suffering from severe malaria as compared to non-severe malaria (*P* < 0.01, Hedges’s g = 3.45, 95% CI 1.06–5.83, *I*^2^ = 98.73, 6 studies). In summary, these findings provide valuable insights into the potential use of UA as a biomarker for malaria infection and determination of its severity. Further research is needed to validate these findings and to explore the underlying mechanisms that contribute to the elevation of UA levels during malaria infection.

## Introduction

Malaria is a significant public health issue, predominantly in tropical and subtropical regions^1^. The World Health Organization reported an estimated 247 million malaria cases worldwide in 2021, causing approximately 625,000 deaths, a 10% increase from 2019^1^. The disease is transmitted via bites from infected female *Anopheles* mosquitoes, which carry various *Plasmodium* species including *P. vivax*, *P. falciparum*, *P. malariae*, *P. ovale*, and *P. knowlesi*^2^. The clinical spectrum of malaria ranges from asymptomatic infections to severe, life-threatening diseases, with severity depending on factors like the *Plasmodium* species, patient immunity, and other host factors^3^. Severe malaria can present with complications such as cerebral malaria, severe anemia, acute kidney injury, and respiratory distress^4^.

Oxidative stress is a significant factor in malaria's symptoms and pathology. Infected red blood cells (RBCs) trigger an immune response, activating immune cells like macrophages and neutrophils, which release reactive oxygen species (ROS) to eliminate infected RBCs^5^. Severe infections, particularly by *P. falciparum* and *P. vivax*, generate substantial oxidative stress, leading to severe damage in multiple organ systems^5,6^. In diagnosing and managing malaria, various biomarkers have been identified to characterize the disease's severity. These biomarkers included C-reactive protein (CRP), Angiopoietin 2 (Ang-2), Ang-2/1 ratio, *Plasmodium falciparum* histidine-rich protein (PfHRP2), and platelet count^7–9^.

Uric acid (UA) is a critical biomarker, being the final product of purine catabolism^10^.

During malaria infection, infected RBCs release hypoxanthine. This hypoxanthine is then converted by the enzyme xanthine dehydrogenase in the bloodstream, an enzyme absent both in the infected RBCs and the *Plasmodium* parasites^11^. Furthermore, UA is associated with oxidative stress. Evidence suggests that UA embodies dual roles: acting as an oxidative stress marker and potentially offering therapeutic benefits as an antioxidant^12,13^. While a multitude of biomarkers provide insights into the status and progression of severe malaria, the significance of UA stands out. Elevated UA levels not only reflect the extent of RBC destruction but also actively influence immune responses and induce inflammation^11,14–16^.

Increased plasma UA levels in malaria patients correlate with a surge in inflammatory cytokines such as tumor necrosis factor (TNF)-α, interleukin (IL)-6, IL-10, and IL-8 in *P. falciparum* patients^17^. This suggests that UA might play a role in triggering various immune cells to release inflammatory cytokines. These inflammatory activities can heighten the symptoms and complications of malaria, underscoring UA's essential role in determining disease severity. It is worth noting that while elevated UA levels are observed in patients with uncomplicated malaria, they are especially pronounced in severe malaria cases^17–20^. By comparing UA levels across these varying disease severities, clinicians can achieve a clearer insight into a patient's disease progression, allowing for more tailored therapeutic strategies^21^. Consequently, this study seeks to elucidate the differences in UA levels between malaria patients and uninfected controls, as well as between patients with severe and non-severe malaria.

## Methods

### Protocol

The systematic review employed the Preferred Reporting Items for Systematic Reviews and Meta-Analyses (PRISMA) as its reporting guideline for systematic reviews and meta-analyses^22^. The protocol of the systematic review was registered at PROPERO (CRD42023431878).

### Search strategy

A comprehensive literature search was carried out across databases such as Embase, MEDLINE, Ovid, PubMed, Scopus, ProQuest, and Google Scholar to identify relevant studies for inclusion. The search was conducted with a combination of keywords and MeSH terms relating to “malaria,” and “uric acid,” as “(urate OR uric OR “uric acid” OR “2,6,8-trihydroxypurine” OR trioxopurine) AND (malaria OR plasmodium OR “Plasmodium Infection” OR “Remittent Fever” OR “Marsh Fever” OR Paludism)” (Table S1). The search spanned studies from inception until June 2023. Additionally, reference lists of included articles were scrutinized to identify potentially relevant studies that were missed during the initial search. Searches on Google Scholar were conducted using the same search strategy as for the main databases to identify potentially relevant studies not indexed there. Only the first 200 articles were screened from the Google Scholar search results for eligibility, as previously recommended^23^.

### Eligibility criteria and study selection

Studies were included if they met the following criteria: (1) observational studies (cross-sectional, case–control, and cohort studies) that reported on UA levels in malaria patients and uninfected controls or in severe and non-severe malaria patients, (2) studies that provided sufficient data (mean/median and standard deviation/interquartile range) to calculate effect sizes, and (3) studies published in English. Reviews, editorials, case reports, and studies lacking full-text availability were excluded.

The study selection process involved two stages. In the initial stage, titles and abstracts of identified studies were independently screened by two reviewers (SK, MK) based on the inclusion and exclusion criteria. In the subsequent stage, full texts of the shortlisted studies were thoroughly assessed for eligibility. Any discrepancies were resolved through discussion, and if required, a third reviewer (KUK) was consulted.

### Data extraction

Data extraction was conducted independently by two reviewers (MK, KUK) using a standardized data extraction form. Any discrepancies were resolved through discussion, and if required, a third reviewer (AM) was consulted. The extracted information from each study encompassed author names, year of publication, study design, geographical continent, age group of participants, species of *Plasmodium*, diagnostic methods for malaria, the type of blood samples collected, and both qualitative and quantitative data pertinent for analysis. It is important to note that while quantitative studies are typically subjected to meta-analysis, qualitative studies more commonly utilize the meta-synthesis or meta-summary approach^24^.

### Quality assessment

The methodological quality of the included studies was evaluated independently by two reviewers (SK, MK) using the JBI critical appraisal tool for observational studies^25^. Studies were assessed based on selection, comparability, and outcome for cohort and case–control studies, and selection, comparability, and exposure for cross-sectional studies. Discrepancies were resolved through discussion, or a third reviewer (KUK) was consulted when necessary.

### Data synthesis and analysis

Thematic synthesis was employed to capture these overarching themes and narratives, synthesizing the collective qualitative insights^26^. The meta-analysis was performed to calculate the pooled effect sizes, expressed as Hedges' g, with 95% confidence intervals (CIs). The Hedges' g was pooled using the random-effects model^27^. Heterogeneity among studies was assessed using the *I*^2^ statistic, with values > 50% indicating substantial heterogeneity^28^. Subgroup analyses were performed based on publication years, study design, geographical continent, age group of participants, species of *Plasmodium*, diagnostic methods for malaria, and the type of blood samples collected. Meta-regression analyses were conducted to explore potential sources of heterogeneity. Sensitivity analysis, or leave-one-out analysis, was conducted to assess the robustness of the results. Publication bias was examined visually using funnel plots and statistically with Egger's test, and the trim-and-fill method was employed to adjust for publication bias. All analyses were performed using Stata software version 17.0, with a *P*-value < 0.05 considered statistically significant, unless otherwise stated.

## Results

### Search results

**Figure 1:**
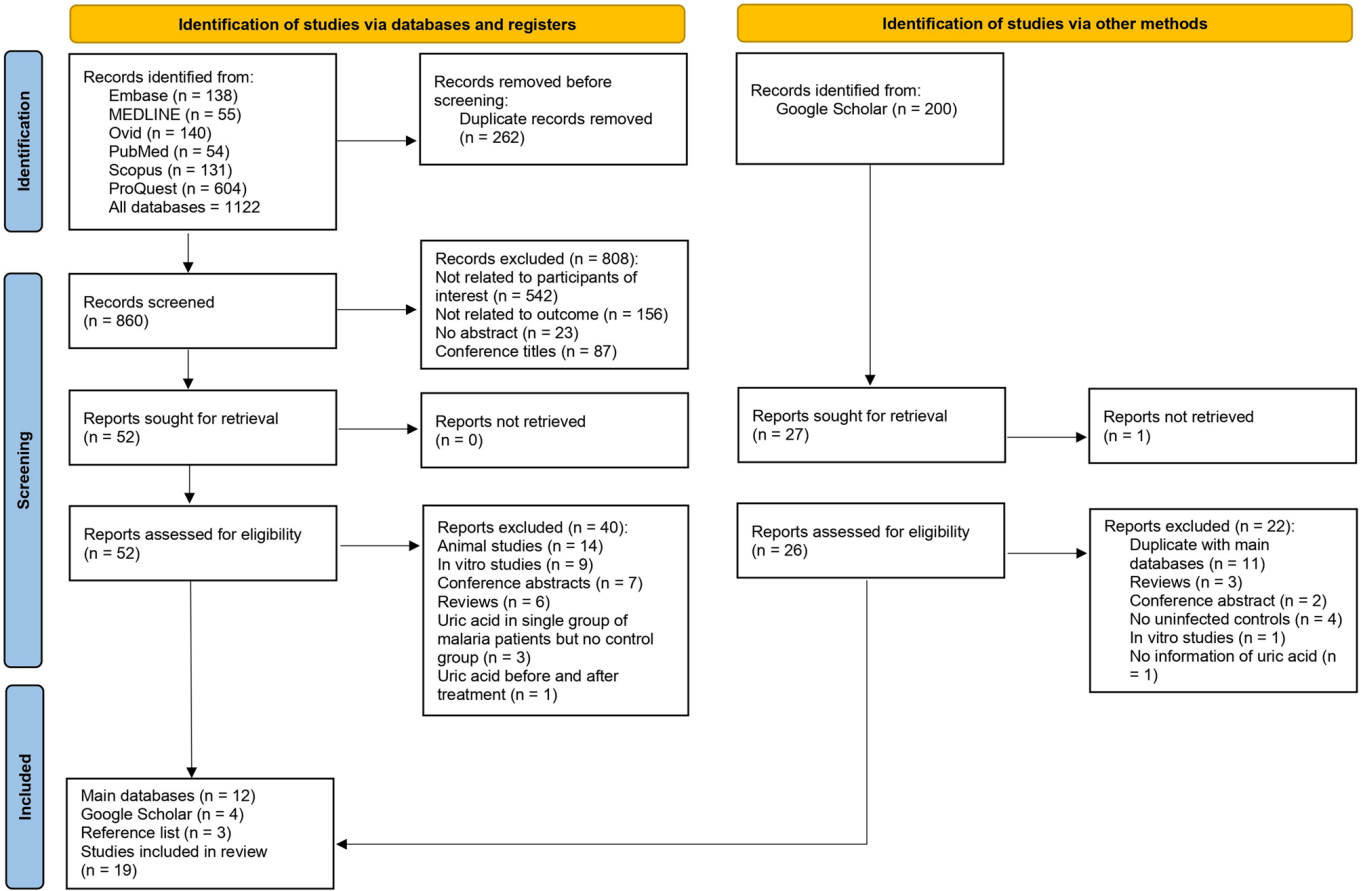
Study flow diagram.

An initial search was conducted across six databases, yielding a total of 1122 articles identified. These consisted of 138 articles from Embase, 55 from MEDLINE, 140 from Ovid, 54 from PubMed, 131 from Scopus, and 604 from ProQuest. Following this, the records were meticulously screened for relevance and suitability, during which a total of 860 articles were examined. This screening process resulted in the exclusion of 808 articles based on specific criteria. The remaining 52 articles were then assessed further for their eligibility. During this stage, 40 articles were excluded due to the nature of the study or its focus, leaving 12 articles for inclusion^4,6,17,19,20,29–35^. The exclusions at this stage were based on the following: 14 were animal studies, nine were in vitro studies, seven were conference abstracts, six were reviews, three were studies on uric acid in a single group of malaria patients but without a control group, and one was a study on uric acid levels before and after treatment. In addition to the initial databases, we also reviewed Google Scholar and reference lists to broaden eligibility criteria. Four studies were identified from Google Scholar^36–39^, and three were identified in reference lists^40–42^, resulting in a final total of 19 studies being included in the review^4,6,17,19,20,29–42^ (Fig. 1).

### Characteristics of studies

This analysis comprised 19 studies, primarily published from 2010 to 2023 (73.7%), and employed cross-sectional (63.2%), cohort (21.1%), and case–control designs (15.8%). The geographical distribution was nearly balanced between Africa (42.1%) and Asia (57.9%), with a preponderance of studies from Nigeria and India. The studies predominantly focused on *P. falciparum* (52.6%) and *P. vivax* (10.5%). A subset of studies investigated multiple *Plasmodium* species, with 15.8% examining both *P. falciparum* and *P. vivax*, and another 10.5% looking into mixed infections involving *P. falciparum*, *P. vivax*, and other species. Participant cohorts ranged across all age groups, with a slightly higher proportion of studies focusing on adults (42.1%). Microscopy was the predominant method for malaria detection (63.2%), with some studies utilizing combined methodologies. The blood samples for uric acid measurement were equally divided between serum and plasma (42.1% each), with some studies unspecified (Table 1, Table S2).

**Table 1:** Characteristics of studies.

Characteristics	No. (19 studies)	%
**Publication year**		
2010–2023	14	73.7
2000–2009	2	10.5
Before 2000	3	15.8
**Study designs**		
Cross-sectional studies	12	63.2
Cohort study	4	21.1
Case–control studies	3	15.8
**Study areas**		
Africa	8	42.1
Nigeria	5	62.5
Cameroon	1	12.5
Ethiopia	1	12.5
Mali	1	12.5
Asia	11	57.9
India	9	81.8
Turkey	1	9.10
Thailand	1	9.10
***Plasmodium*** **species**		
* P. falciparum*	10	52.6
* P. falciparum, P. vivax*	3	15.8
* P. falciparum, P. vivax,* mixed infections	2	10.5
* P. vivax*	2	10.5
Not specified	2	10.5
**Participants**		
Children	6	31.6
Adults	8	42.1
All age groups	2	10.5
Not specified	3	15.8
**Methods for** ***Plasmodium*** **detection**		
Microscopy	12	63.2
Microscopy/RDT	4	21.1
Microscopy/QBC/RDT	1	5.26
Not specified	2	10.5
**Blood sample for UA measurement**		
Serum	8	42.1
Plasma	8	42.1
Not specified	3	15.8

*UA* uric acid, *RDT* rapid diagnostic test, *QBT* quantitative buffy coat assay.

### Quality of studies

The quality of the studies was assessed using the JBI critical appraisal checklist for observational studies. Among the cross-sectional studies, consistent adherence to the checklist outcomes was observed in the studies conducted by Aqeel et al*.*^29,30^, Ayede et al.^31^, Bertrand et al.^20^, Bhardwaj et al.^32^, Karua and Kishan^41^, Okon et al.^34^, Prabhu et al.^42^, and Iwalokun et al.^19^. However, some studies such as Erel et al.^38^, Idonije et al*.*^4^, and Thurnham et al.^35^ had an unclear status regarding the inclusion criteria and addressing confounding factors. Among the case–control studies, Ebrahim et al.^37^, Hatim et al.^40^, and Olisekodiaka et al.^39^ lacked identification of confounding factors and strategies to address them. In the cohort studies, Chandrashekhar et al.^33^ and Lopera-Mesa et al.^17^ lacked identification of confounding factors, strategies to address them, and details on follow-up time. Das et al.^36^ study also lacked identification of confounding factors, strategies to address them, and details on follow-up time. Moreover, Das BS and Nanda NK^6^ study was unclear regarding the utilization of strategies to address incomplete follow-up (Table S3).

### Qualitative synthesis of UA levels

From thematic synthesis, the predominant theme that emerged from studies was the general consensus that malaria patients exhibited higher UA levels compared to uninfected controls^6,19,20,30,32–34,37–40,42^. This observation was further supported by narratives and discussions emphasizing the clinical implications of these elevated levels. Furthermore, several studies emphasized an intensified theme revolving around even higher UA levels in severe malaria cases compared to non-severe ones^17,19,30,33,34,41^. An interesting narrative thread found in the four studies posits a direct relationship between the intensity of malaria parasitemia and rising UA levels^17,31,34,39^. However, contrasting themes were evident in studies by Aqeel et al.^29^ and Idonije et al.^4^, where narratives did not underscore a significant difference in UA levels between the two groups (Table 2).

**Table 2:** Outcomes of included studies.

No	Authors	Study location	*Plasmodium* spp.	Age range (years)	Qualitative synthesis of UA
1	Aqeel et al., 2019	India	*P. falciparum/P. vivax*	Not specified	1. No difference in UA levels between malaria and uninfected controls. 2. No difference in UA levels between *P. falciparum* and *P. vivax* malaria
2	Aqeel et al., 2021	India	*P. vivax*	> 15 years	1. UA levels were higher in malaria than uninfected controls. 2. UA levels were higher in severe than non-severe malaria
3	Ayede et al., 2017	Nigeria	*P. falciparum*	2–15 years	1. No difference in UA levels between malaria and uninfected controls. 2. There was a positive relationship between malaria parasitemia and UA levels
4	Bertrand et al., 2008	Cameroon	*P. falciparum*	15–79 years	UA levels were higher in malaria than uninfected controls
5	Bhardwaj et al., 2020	India	*P. falciparum*	Not specified	1. UA levels were higher in malaria than uninfected controls. 2. UA levels were lower in severe than non-severe malaria
6	Chandrashekhar et al., 2022	India	*P. falciparum/P. vivax/*mixed infections	18–40 years	1. UA levels were higher in malaria than uninfected controls. 2. UA levels were higher in severe than non-severe malaria. 3. No difference in UA levels between *P. falciparum* and *P. vivax* malaria. 4. *P. falciparum* had higher UA levels than mixed infection but no difference in UA levels between *P. falciparum* and *P. vivax*
7	Das BS and Nanda NK, 1999	India	*P. falciparum*	2–12 years	UA levels were higher in malaria than uninfected controls (slight difference but may not be significant)
8	Das et al., 2022	India	*P. falciparum/P. vivax*	18–65 years	UA levels were increased in 18.51% of patients with severe malaria compared to only 4.91% with non-severe malaria (*P* = 0.04)
9	Ebrahim et al., 2019	Ethiopia	*P. falciparum*	Not specified	UA levels were higher in malaria than uninfected controls
10	Erel et al., 1997	Turkey	*P. vivax*	15–35 years	UA levels were higher in malaria than uninfected controls
11	Hatim et al., 2022	India	*P. falciparum/P. vivax/*mixed infections	20–45 years	UA levels were higher in malaria than uninfected controls
12	Idonije et al., 2011	Nigeria	Not specified	Not specified	No difference in UA levels between malaria and uninfected controls
13	Iwalokun et al., 2006	Nigeria	*P. falciparum*	3–12 years	1. UA levels were higher in malaria (asymptomatic, uncomplicated, severe malaria) than uninfected controls. 2. UA levels were higher in severe than non-severe malaria
14	Karua PC and Kishan M, 2020	India	*P. falciparum/P. vivax*	> 15 years	UA levels were higher in severe than non-severe malaria
15	Lopera-Mesa et al., 2012	Mali	*P. falciparum*	6 months–17 years	1. UA levels were higher in severe than non-severe malaria. 2. There was a positive relationship between malaria parasitemia and UA levels
16	Okon et al., 2022	Nigeria	*P. falciparum*	≤ 15 years	1. UA levels were higher in malaria than uninfected controls. 2. UA levels were higher in severe than non-severe malaria. 3. There was a positive relationship between malaria parasitemia and UA levels
17	Olisekodiaka et al., 2017	Nigeria	*P. falciparum*	1–5 years	1. UA levels were higher in malaria than uninfected controls. 2. There was a positive relationship between malaria parasitemia and UA levels
18	Prabhu et al., 2021	India	Not specified	Not specified	UA levels were higher in malaria than uninfected controls
19	Thurnham et al., 1990	Thailand	*P. falciparum*	6–38 years	Not specified

*UA* uric acid.

### UA levels in patients suffering from malaria as compared to uninfected controls

**Figure 2:**
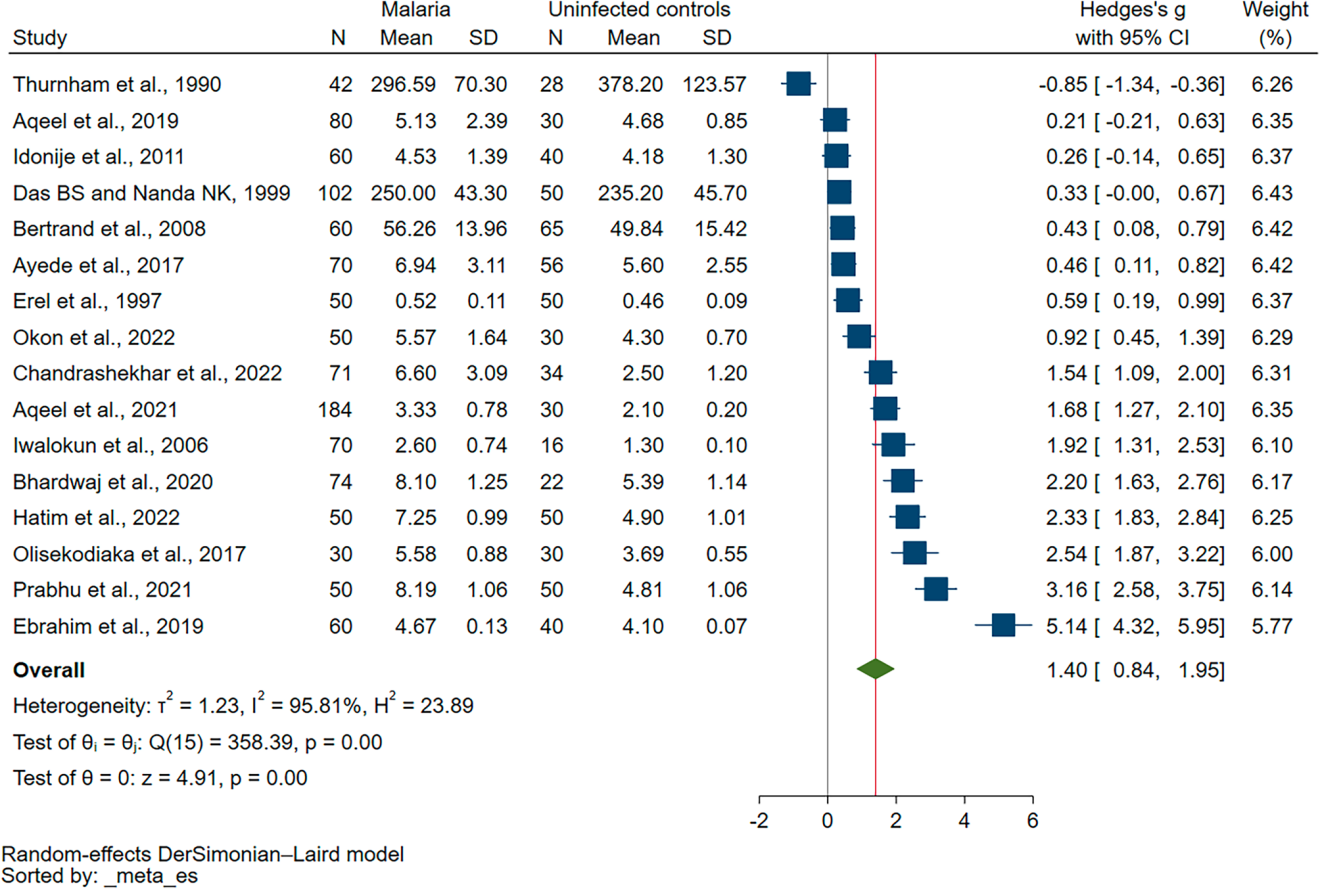
The forest plot shows the difference in the uric levels between malaria patients and uninfected controls. *CI* confidence interval, *N* number of participants, *SD* standard deviation.

In the quantitative synthesis, sixteen studies were deemed eligible for inclusion, owing to the presence of quantitative data^4,6,19,20,29–35,37–40,42^. The conducted meta-analysis demonstrated a significant elevation in UA levels in patients suffering from malaria as compared to uninfected controls (*P* < 0.01, Hedges’s g = 1.40, 95% CI 0.84–1.95, *I*^2^ = 95.81, 16 studies, Fig. 2). Furthermore, meta-regression analyses were performed, factoring in publication years, study design, geographical continent, age group of participants, species of *Plasmodium*, diagnostic methods for malaria, and the type of blood samples collected. The analyses demonstrated that both the year of publication and the employed study design significantly influenced the pooled effect estimate (*P* < 0.05, refer to Table S4). This evidence suggests that these two variables should be given due consideration while interpreting the results.

The subgroup analyses of UA levels between malaria cases and uninfected controls have been conducted. For studies published between 2010 and 2023, a statistically significant higher UA level in malaria patients (*P* < 0.01, Hedges’ g = 1.93, 95% CI 1.11–2.54, *I*^2^ = 95.88) was identified across 11 studies. However, studies published before 2010 did not present a statistically significant difference. In terms of study design, both cross-sectional and case–control studies showed significantly higher UA levels in malaria cases (*P* < 0.01), while cohort studies did not. Geographically, studies from both Africa and Asia consistently revealed higher UA levels in malaria cases (*P* < 0.01). When assessing age groups, children and adults had significantly higher UA levels (*P* < 0.01), while groups with unspecified age or encompassing all age groups did not demonstrate statistical significance. UA levels varied across different *Plasmodium* species, with significantly higher UA levels identified in *P. falciparum* and mixed infection cases (*P* < 0.01). Cases with *P. vivax* also had higher UA levels, but the significance level was less robust (*P* = 0.04). The diagnostic method for malaria (microscopy or microscopy/RDT) and types of blood samples (serum or plasma) also showed significant differences in UA levels. However, UA levels in cases where diagnostic methods and types of blood samples were not specified did not reach statistical significance (Table 3).

**Table 3:** Subgroup analyses of UA levels between malaria cases and uninfected controls.

Subgroup analyses	*P* value	Hedges’ g (95% CI)	*I*^*2*^ (%)	Number of studies
Publication years				
2010–2023	< 0.01	1.93 (1.11–2.54)	95.88	11
2000–2009	0.12	1.15 (− 0.30 to 2.61)	94.14	2
Before 2000	0.91	0.04 (− 0.73 to 0.81)	90.75	3
Study design				
Cross-sectional study	< 0.01	0.98 (0.41–1.55)	94.53	11
Case–control study	< 0.01	3.31 (1.73–4.89)	94.25	3
Cohort study	0.12	0.93 (− 0.26 to 2.11)	94.26	2
Continent				
Africa	< 0.01	1.62 (0.70–2.54)	96.24	7
Asia	< 0.01	1.23 (0.49–1.98)	95.97	9
Age group				
Children	< 0.01	1.19 (0.47–1.91)	91.90	5
Adults	< 0.01	1.45 (0.08–2.09)	92.33	6
All age groups	0.48	2.13 (− 3.73 to 8.00)	99.34	2
Not specified age	0.15	1.20 (− 0.45 to 2.84)	97.41	3
*Plasmodium* species				
* P. falciparum*	< 0.01	1.42 (0.58–2.25)	96.50	9
* P. vivax*	0.04	1.14 (0.07–2.20)	92.74	2
* P. falciparum, P. vivax,* mixed infections	< 0.01	1.96 (1.16–2.70)	80.55	2
* P. falciparum, P. vivax*	N/A	N/A	N/A	1
Not specified	0.24	1.70 (− 1.15 to 4.55)	98.45	2
Diagnostic method for malaria				
Microscopy	< 0.01	1.42 (0.70–2.13)	96.18	11
Microscopy/RDT	0.02	1.14 (0.21–2.08)	92.99	3
Not specified	0.24	1.70 (− 1.15 to 4.55)	98.45	2
Types of blood samples				
Serum	< 0.01	1.82 (0.8–2.84)	96.28	6
Plasma	0.03	0.82 (0.09–1.56)	94.72	7
Not specified	0.03	1.93 (0.16–3.70)	97.67	3

*CI* confidence interval, *N/A* not assessed, *RDT* rapid diagnostic test.

### UA levels in patients suffering from severe malaria as compared to non-severe malaria

**Figure 3:**
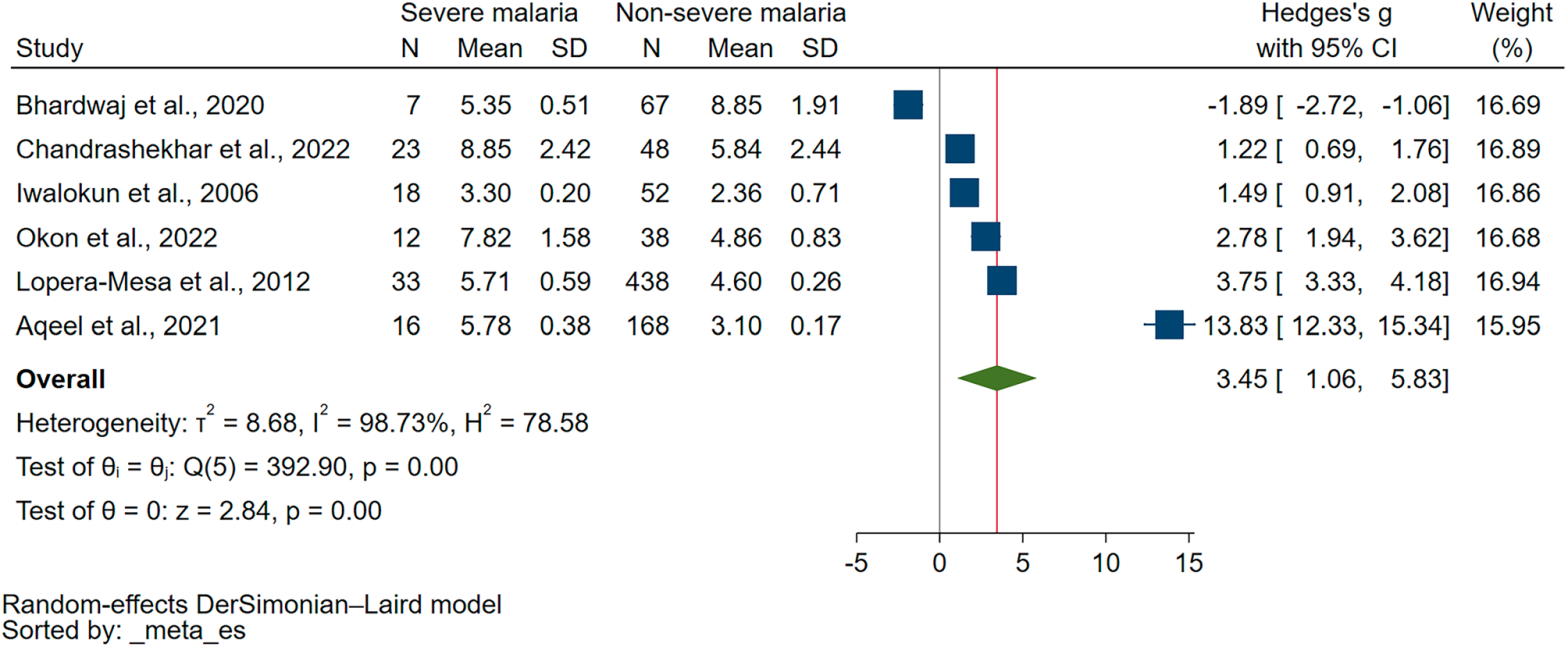
The forest plot shows the difference in the uric levels between patients with severe malaria and those with non-severe malaria. *CI* confidence interval, *N* number of participants, *SD* standard deviation.

In the quantitative synthesis, six studies were deemed eligible for inclusion, owing to the presence of quantitative data^17,19,30,32–34^. The conducted meta-analysis demonstrated a significant elevation in UA levels in patients suffering from severe malaria as compared to non-severe malaria (*P* < 0.01, Hedges’s g = 3.45, 95% CI = 1.06–5.83, *I*^*2*^ = 98.73, 6 studies, Fig. 3). Furthermore, meta-regression analyses were performed, factoring in publication years, study design, geographical continent, age group of participants, species of *Plasmodium*, diagnostic methods for malaria, and the type of blood samples collected. The analyses divulged that none of these factors significantly influenced the pooled effect estimate (*P* > 0.05, Table S4).

The subgroup analysis comparing UA levels between severe and non-severe malaria cases presents insightful results. For the publication years 2010–2023, UA levels were significantly higher in severe malaria patients (*P* = 0.01, Hedges’ g = 3.87, 95% CI 0.87–6.87, *I*^2^ = 98.95%, 5 studies). However, data for the years 2000–2009 were not applicable. In terms of study design, cohort studies showed higher UA levels in severe cases with marginal significance (*P* = 0.05, Hedges’ g = 2.49, 95% CI 0.02–4.97, *I*^2^ = 98.11%, 2 studies), while cross-sectional studies did not reach statistical significance (*P* = 0.08, Hedges’ g = 4.00, 95% CI − 0.44 to 8.44, *I*^2^ = 99.09%, 4 studies). Regionally, higher UA levels in severe cases were significant in Africa (*P* < 0.01, Hedges’ g = 2.68, 95% CI 1.19–4.17, *I*^2^ = 94.68%, 3 studies), while the significance was not reached in Asia (*P* = 0.18, Hedges’ g = 4.34, 95% CI − 1.98 to 10.67, *I*^2^ = 99.38%, 3 studies). In terms of age groups, significantly higher UA levels in severe cases were found in both children and adults, but with a higher degree of heterogeneity in the adult subgroup. Among the *Plasmodium* species, data for *P. falciparum* were applicable, but the results were not statistically significant. Similarly, the diagnostic method used for malaria did not significantly influence the UA levels between the groups. In terms of blood sample types, serum samples showed significant differences (*P* = 0.03, Hedges’ g = 4.23, 95% CI 0.40–8.05, *I*^2^ = 99.19%, 4 studies), while plasma samples also reached statistical significance (*P* = 0.01, Hedges’ g = 1.97, 95% CI 0.44–3.49, *I*^*2*^ = 89.35%, 2 studies, Table 4).

**Table 4:** Subgroup analyses of UA levels between severe and non-severe malaria.

Subgroup analyses	*P* value	Hedges’ g (95% CI)	*I*^*2*^ (%)	Number of studies
Publication years				
2010–2023	0.01	3.87 (0.87–6.87)	98.95	5
2000–2009	N/A	N/A	N/A	1
Study design				
Cross-sectional study	0.08	4.00 (− 0.44 to 8.44)	99.09	4
Cohort study	0.05	2.49 (0.02–4.97)	98.11	2
Continent				
Africa	< 0.01	2.68 (1.19–4.17)	94.68	3
Asia	0.18	4.34 (− 1.98 to 10.67)	99.38	3
Age group				
Children	< 0.01	2.68 (1.19–4.17)	94.68	3
Adults	0.18	4.34 (− 1.98 to 10.67)	99.38	3
*Plasmodium* species				
*P. falciparum*	0.18	1.55 (− 0.71 to 3.80)	98.02	4
* P. vivax*	N/A	N/A	N/A	1
* P. falciparum, P. vivax,* mixed infections	N/A	N/A	N/A	1
Diagnostic method for malaria				
Microscopy	0.18	1.55 (− 0.71 to 3.80)	98.02	4
Microscopy/RDT	0.23	7.51 (− 4.85 to 19.86)	99.58	2
Types of blood samples				
Serum	0.03	4.23 (0.40–8.05)	99.19	4
Plasma	0.01	1.97 (0.44–3.49)	89.35	2

*CI* confidence interval, *N/A* not assessed, *RDT* rapid diagnostic test.

### Sensitivity analysis

**Figure 4:**
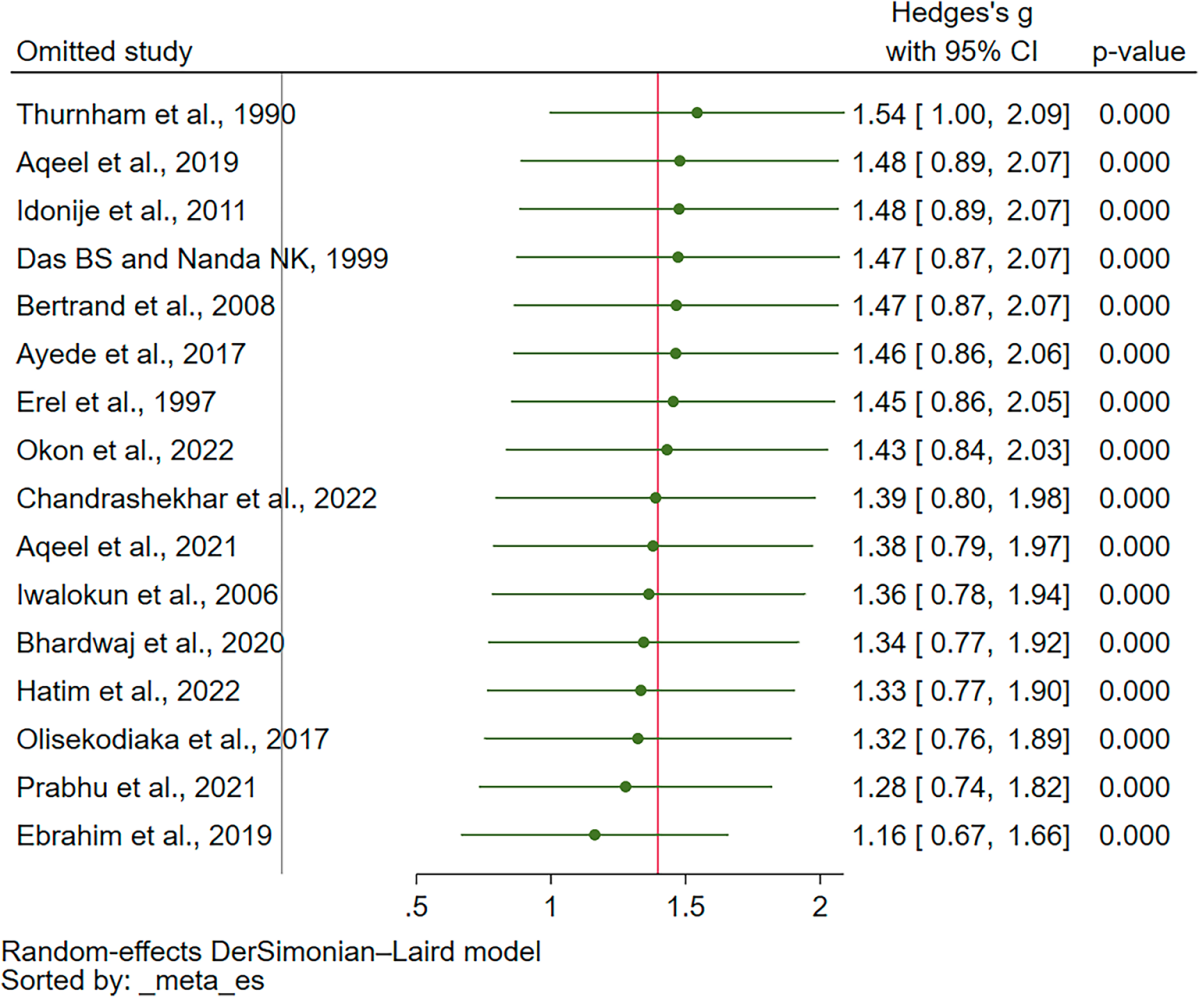
The leave-one-out method showed the re-run meta-analysis of the difference in the uric levels between malaria patients and uninfected controls. *CI* confidence interval.

The robustness of the meta-analysis was evaluated using a leave-one-out analysis, which tests the influence of individual studies on the overall meta-analysis result by systematically removing each study and re-running the analysis. When applied to the comparison of UA levels between malaria patients and uninfected controls, this analysis revealed consistently significant elevations in UA levels in malaria patients across all re-run analyses (*P* < 0.05 in all cases, as depicted in Fig. 4). These results demonstrate the robustness of the meta-analysis findings, as the overall significance was maintained irrespective of the exclusion of any single study.

**Figure 5:**
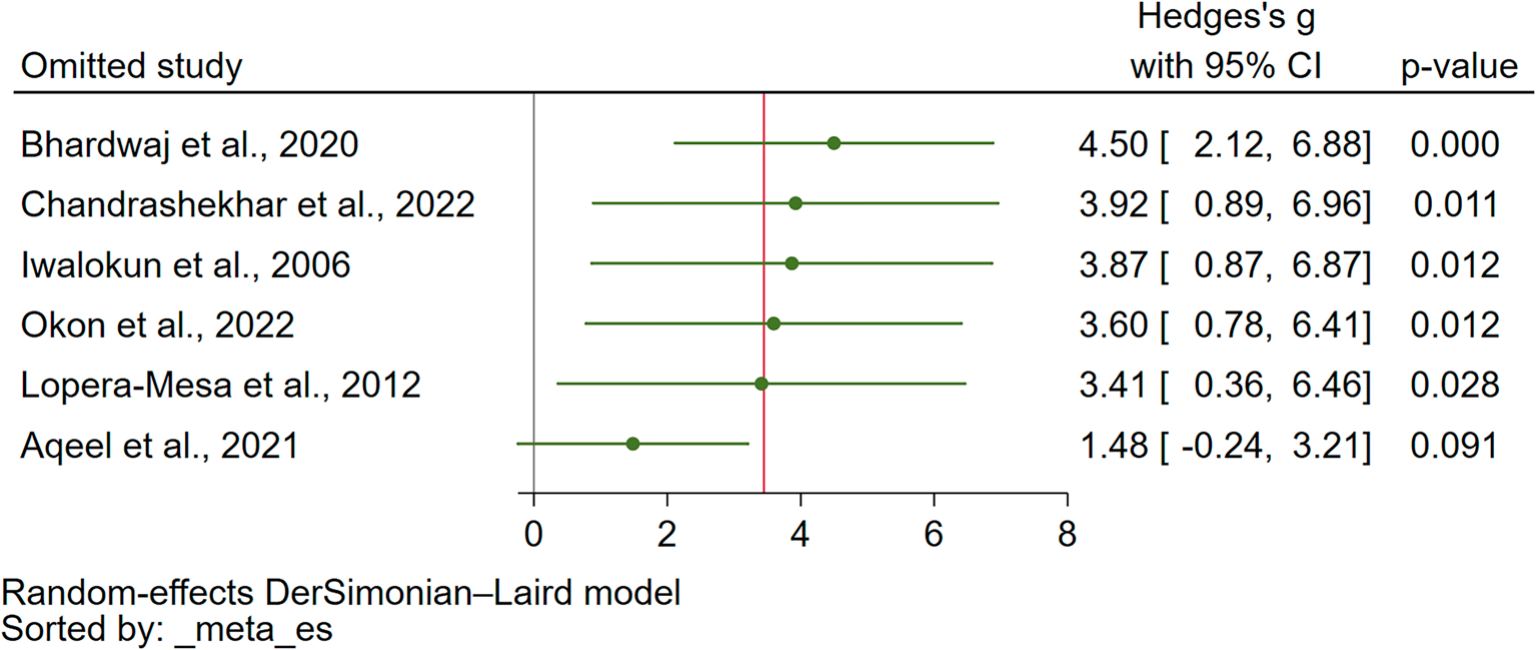
The leave-one-out method showed the re-run meta-analysis of the difference in the uric levels between patients with severe and non-severe malaria. *CI* confidence interval.

On the other hand, the results were different when this leave-one-out analysis was applied to the comparison of UA levels between severe and non-severe malaria cases. In this case, the significance of the results varied depending on the study that was excluded (as shown in Fig. 5, with *P* < 0.05 in some reruns and *P* > 0.05 in others). This variation in results indicates that the meta-analysis outcomes in this context were not robust, and the overall conclusions were sensitive to the removal of individual studies.

### Publication bias

**Figure 6:**
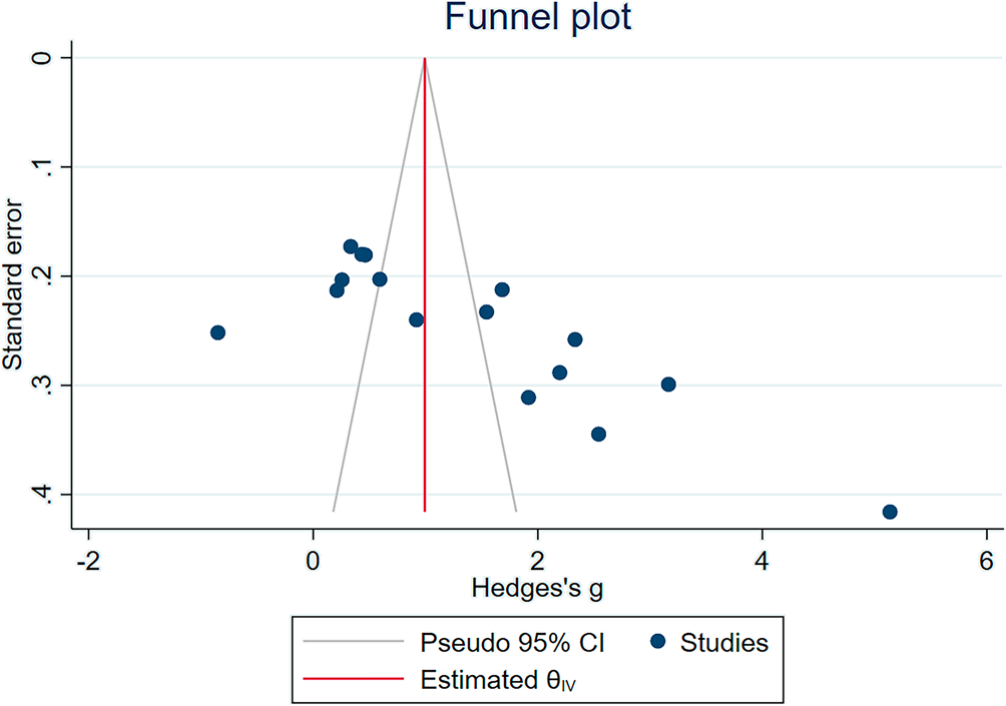
The funnel plot showing an asymmetrical distribution of the effect estimates between the middle line of the plot. *CI* confidence interval.

Visual inspection of the funnel plot unveiled an asymmetrical distribution (as depicted in Fig. 6), suggesting potential bias in the meta-analysis comparing UA levels between malaria cases and uninfected controls. Further substantiating this, Egger’s test revealed a significant small-study effect (*P* < 0.01), indicating the presence of publication bias likely attributable to the disproportionate influence of smaller studies.

To correct for this observed bias, the trim-and-fill method was implemented. This statistical technique adjusts the overall effect size by estimating and correcting for the number and outcomes of hypothetically missing studies due to publication bias. After applying this method, the revised results continued to indicate a significant increase in UA levels among malaria patients compared to uninfected controls (Table S5).

## Discussion

The study results shed light on the intricate link between UA levels and malaria, and underscore the significance of certain study-related variables, such as the year of publication and study design, in determining the strength and direction of this association. A meta-analysis of 16 studies revealed a significant elevation in UA levels in patients afflicted with malaria as compared to uninfected controls. Notably, the year of publication and the employed study design emerged as significant factors influencing the pooled effect estimate. This raises important questions about potential changes in research methodology, laboratory techniques, or other unmeasured confounding variables that might have changed over the years and could affect the reliability of the association between UA levels and malaria. Furthermore, the results of the subgroup analyses show nuances in UA levels between malaria cases and uninfected controls. Studies conducted more recently (2010–2023) found a significant increase in UA levels among malaria patients, whereas older studies did not. It is possible that recent improvements in diagnostic and analytical techniques might have contributed to these results. As for the study design, both cross-sectional and case–control studies revealed a significant increase in UA levels in malaria cases, suggesting that these designs might be more sensitive at detecting this difference. The meta-analysis also reveals that the geographic location and age group of the participants play a crucial role in determining the UA levels. Studies conducted in Africa and Asia consistently showed elevated UA levels in malaria patients, which could reflect the high malaria burden in these regions^1^.

Interestingly, UA levels varied between *P. falciparum* and *P. vivax* infections. This variability could be attributed to the unique pathogenesis and disease manifestations between *P. falciparum* and *P. vivax* infections^43,44^. The fact that cases of *P. falciparum* and mixed infections exhibited significantly higher UA levels suggests a potentially more severe inflammatory response associated with these types of infections. For different age groups, both adult and pediatric patients had higher UA levels, underscoring the potential of UA as a biomarker for malaria across all age groups. Nevertheless, UA may be used as a biomarker for malaria severity only in children because there was no difference  in UA between severe and non-severe malaria among studies that enrolled adults.

The current meta-analysis included six studies due to the availability of quantitative data^17,19,30,32–34^, underscored a significant increase in UA levels in patients with severe malaria compared to those with non-severe malaria. Elevated UA levels appear to be associated with severe malaria; however, this association seems to be influenced by factors such as geographical location and the age group of participants, as demonstrated in the subgroup meta-analysis. Given the observed heterogeneity among the studies reviewed, the findings from the meta-analysis should be approached with caution. The diversity in methodologies and objectives of the primary studies inherently adds complexity to a comprehensive interpretation. While this review identifies a potential link between UA levels and malaria severity, it is essential for readers and researchers to contextualize each primary study's aims and methods when drawing conclusions from these insights. Moreover, delving deeper into the mechanisms underlying this association is vital, not only for a clearer understanding of malaria's pathophysiology but also for uncovering potential therapeutic targets, enhancing predictions of disease severity, and tailoring treatments more effectively.

Previous in vitro investigations have demonstrated that the induction of the innate immune response to *Plasmodium* infection, specifically in human dendritic cells, is facilitated by the presence of UA precipitates derived from *P. falciparum*. This process involves the maturation of dendritic cells, leading to elevated expression levels of cell surface co-stimulatory molecules such as CD40, CD80, and CD86^45,46^. In a similar vein, a reduction in UA levels in vivo has the potential to mitigate the host's inflammatory response and alleviate associated pathology^46^. A previous study revealed that the formation of UA through the degradation of *Plasmodium*-derived hypoxanthine/xanthine triggers the release of TNF, which is a cytokine associated with the response to *Plasmodium* infections^47^. Moreover, inhibiting the formation of UA significantly diminishes the secretion of cytokines such as TNF, IL-6, IL-1β, and IL-10, which are known to be involved in the response to *P. falciparum* infections and are associated with the pathogenesis of malaria^48^. Another study indicated that patients with *P. vivax* malaria exhibit lower levels of uric acid during the acute phase compared to the convalescent phase, suggesting clinical improvement resulting from antimalarial treatment or as a negative finding in *P. vivax* malaria^49^. Conversely, it has been reported that UA levels increase during the acute phase of *P. falciparum* malaria and correlate with disease severity^17^. The elevated levels of UA contribute to the pathogenesis of *P. falciparum* malaria by inducing endothelial pathology, as evidenced by the basal plasma levels of UA and endothelial biomarkers, which rise during episodes of uncomplicated malaria and escalate further with increasing disease severity^50^.

The present study is subject to certain limitations. Firstly, there was a publication bias detected in the meta-analysis results, which may lead to an overestimation of the effect size. Secondly, our meta-analysis demonstrated a high *I*^*2*^ value, indicating substantial heterogeneity among the included studies. This heterogeneity, resulting from differences in study design, population demographics, geographical location, diagnostic methods, and other factors, complicates the task of drawing definitive conclusions. Thirdly, although our meta-regression analyses factored in several variables, there may still be unconsidered confounding factors that could potentially influence the observed relationship between malaria severity and UA levels. Fourthly, the limited number of studies included in our analysis constrains the breadth of our conclusions, making it challenging to generalize across all malaria patients or geographical locations. Lastly, the limited data available for both severe and non-severe malaria patients may impinge on the reliability of the comparison made between these two groups. Consequently, future research should aim to address these limitations for a more comprehensive understanding of the topic.

## Conclusion

In light of the objective to synthesize the difference in UA levels between malaria patients and uninfected controls, as well as between patients with varying malaria severity, the review found evidence supporting the potential role of UA as a distinguishing biomarker. Elevated UA levels were observed more prominently in malaria patients, especially those with severe manifestations, when compared to uninfected controls. However, it is crucial to approach these findings with caution due to certain study-related variables that could influence results. While the findings underscore UA's potential as an indicative marker for malaria infection and its severity, further research is imperative to validate these observations and delve deeper into the mechanisms prompting the surge in UA levels during a malaria infection.

### Supplementary Information

Supplementary Table 1.

Supplementary Table 2.

Supplementary Table 3.

Supplementary Table 4.

Supplementary Table 5.

## Data Availability

All data relating to the present study are available in this manuscript and supplementary files.
