# Supplementary material for: Association of uric acid levels with severity of Plasmodium infections: a systematic review and meta-analysis
Source: Sci Rep. 2023 Sep 11;13:14979. doi: 10.1038/s41598-023-42217-8 (PMC10495360; doi:10.1038/s41598-023-42217-8)
Supplement: Supplementary file 1 — Supplementary Table 1. [file 41598_2023_42217_MOESM1_ESM.docx]

**Association of uric acid levels with severity of *Plasmodium* infections: A systematic review and meta-analysis**

Saruda Kuraeiad^1^, Kwuntida Uthaisar Kotepui^1^, Frederick Ramirez Masangkay^2^, Aongart Mahittikorn^3^, Manas Kotepui^1^*

^1^Medical Technology, School of Allied Health Sciences, Walailak University, Tha Sala, Nakhon Si Thammarat, Thailand

^2^Department of Medical Technology, Faculty of Pharmacy, University of Santo Tomas, Manila, Philippines

^3^Department of Protozoology, Faculty of Tropical Medicine, Mahidol University, Bangkok, Thailand

**^*^Corresponding author**

Manas Kotepui: manas.ko@wu.ac.th

Saruda Kuraeiad: [saruda.ku@wu.ac.th](mailto:saruda.ku@wu.ac.th)

Frederick Ramirez Masangkay: frederick_masangkay2002@yahoo.com

Aongart Mahittikorn: [aongart.mah@mahidol.ac.th](mailto:aongart.mah@mahidol.ac.th)

Kwuntida Uthaisar Kotepui: [kwuntida.ut@wu.ac.th](mailto:kwuntida.ut@wu.ac.th)

**General keywords**

(urate OR uric OR “uric acid” OR “2,6,8-trihydroxypurine” OR trioxopurine) AND (malaria OR plasmodium OR “Plasmodium Infection“ OR “Remittent Fever“ OR “Marsh Fever“ OR Paludism)

PubMed 1 June 2023

| No. | Key concept | Search terms | Results |
| --- | --- | --- | --- |
| 1. | Uric acid | “uric acid”[Text Word] OR “uric acid”[MeSH Terms] OR urate[Text Word] OR urate[MeSH Terms] OR “2,6,8-trihydroxypurine”[Text Word] OR “2,6,8-trihydroxypurine”[MeSH Terms] OR trioxopurine[Text Word] OR trioxopurine[MeSH Terms] | 49,274 |
| 2. | Malaria | malaria[Text Word] OR plasmodium[Text Word] OR “Infections, Plasmodium“[MeSH Terms] OR “Infection, Plasmodium“[MeSH Terms] OR “Plasmodium Infection“[MeSH Terms] OR “Remittent Fever“[MeSH Terms] OR “Fever, Remittent“[MeSH Terms] OR “Marsh Fever“[MeSH Terms] OR “Fever, Marsh“[MeSH Terms] OR Paludism [MeSH Terms] | 118,041 |
| 3. | 1 AND 2 | (“uric acid”[Text Word] OR “uric acid”[MeSH Terms] OR urate[Text Word] OR urate[MeSH Terms] OR “2,6,8-trihydroxypurine”[Text Word] OR “2,6,8-trihydroxypurine”[MeSH Terms] OR trioxopurine[Text Word] OR trioxopurine[MeSH Terms]) AND malaria[Text Word] OR plasmodium[Text Word] OR “Infections, Plasmodium“[MeSH Terms] OR “Infection, Plasmodium“[MeSH Terms] OR “Plasmodium Infection“[MeSH Terms] OR “Remittent Fever“[MeSH Terms] OR “Fever, Remittent“[MeSH Terms] OR “Marsh Fever“[MeSH Terms] OR “Fever, Marsh“[MeSH Terms] OR Paludism [MeSH Terms] | 54 |

Embase 1 June 2023

| No. | Key concept | Search terms | Results |
| --- | --- | --- | --- |
| 1. | Uric acid | “uric acid”:ti,ab,kw,de OR “uric acid”/exp OR urate:ti,ab,kw,de OR urate/exp OR “2,6,8-trihydroxypurine”:ti,ab,kw,de OR “2,6,8-trihydroxypurine”/exp OR trioxopurine:ti,ab,kw,de OR trioxopurine/exp | 82818 |
| 2. | Malaria | malaria:ti,ab,kw,de OR plasmodium:ti,ab,kw,de OR ‘Remittent Fever’:ti,ab,kw,de OR ‘Marsh Fever’:ti,ab,kw,de OR Paludism:ti,ab,kw,de OR malaria/exp | 156,044 |
| 3. | 1 AND 2 | (“uric acid”:ti,ab,kw,de OR “uric acid”/exp OR urate:ti,ab,kw,de OR urate/exp OR “2,6,8-trihydroxypurine”:ti,ab,kw,de OR “2,6,8-trihydroxypurine”/exp OR trioxopurine:ti,ab,kw,de OR trioxopurine/exp) AND (malaria:ti,ab,kw,de OR plasmodium:ti,ab,kw,de OR ‘Remittent Fever’:ti,ab,kw,de OR ‘Marsh Fever’:ti,ab,kw,de OR Paludism:ti,ab,kw,de OR malaria/exp) | 138 |

Scopus 1 June 2023

| No. | Key concept | Search terms | Results |
| --- | --- | --- | --- |
| 1. | Uric acid | TITLE-ABS-KEY (urate OR uric OR “uric acid” OR “2,6,8-trihydroxypurine” OR trioxopurine) | 81,390 |
| 2. | Malaria | TITLE-ABS-KEY ( ( malaria OR plasmodium OR "plasmodium infection" OR "remittent fever" OR "marsh fever" OR paludism ) ) | 156,710 |
| 3. | 1 AND 2 | ( TITLE-ABS-KEY ( urate OR uric OR "uric acid" OR "2,6,8-trihydroxypurine" OR trioxopurine ) ) AND ( TITLE-ABS-KEY ( ( malaria OR plasmodium OR "Plasmodium Infection" OR "Remittent Fever" OR "Marsh Fever" OR paludism ) ) ) | 131 |

MEDLINE 1 June 2023

| No. | Key concept | Search terms | Results |
| --- | --- | --- | --- |
| 1. | Uric acid AND Malaria | (urate OR uric OR “uric acid” OR “2,6,8-trihydroxypurine” OR trioxopurine) AND (malaria OR plasmodium OR “Plasmodium Infection“ OR “Remittent Fever“ OR “Marsh Fever“ OR Paludism) | 55 |

Ovid 1 June 2023

| No. | Key concept | Search terms | Results |
| --- | --- | --- | --- |
| 1. | Uric acid AND Malaria | (urate OR uric OR “uric acid” OR “2,6,8-trihydroxypurine” OR trioxopurine) AND (malaria OR plasmodium OR “Plasmodium Infection“ OR “Remittent Fever“ OR “Marsh Fever“ OR Paludism) {No Related Terms} limit to (ovid full text available and articles with abstracts and original articles) | 140 |

ProQuest 1 June 2023

| No. | Key concept | Search terms | Results |
| --- | --- | --- | --- |
| 1. | Uric acid AND Malaria | (urate OR uric OR “uric acid” OR “2,6,8-trihydroxypurine” OR trioxopurine) AND (malaria OR plasmodium OR “Plasmodium Infection“ OR “Remittent Fever“ OR “Marsh Fever“ OR Paludism) | 604 |
