# Supplementary material for: Association of uric acid levels with severity of Plasmodium infections: a systematic review and meta-analysis
Source: Sci Rep. 2023 Sep 11;13:14979. doi: 10.1038/s41598-023-42217-8 (PMC10495360; doi:10.1038/s41598-023-42217-8)
Supplement: Supplementary file 4 — Supplementary Table 4. [file 41598_2023_42217_MOESM4_ESM.docx]

**Association of uric acid levels with severity of *Plasmodium* infections: A systematic review and meta-analysis**

Saruda Kuraeiad^1^, Kwuntida Uthaisar Kotepui^1^, Frederick Ramirez Masangkay^2^, Aongart Mahittikorn^3*^, Manas Kotepui^1^*

^1^Medical Technology, School of Allied Health Sciences, Walailak University, Tha Sala, Nakhon Si Thammarat, Thailand

^2^Department of Medical Technology, Faculty of Pharmacy, University of Santo Tomas, Manila, Philippines

^3^Department of Protozoology, Faculty of Tropical Medicine, Mahidol University, Bangkok, Thailand

**^*^Corresponding author**

Manas Kotepui: manas.ko@wu.ac.th

Saruda Kuraeiad: [saruda.ku@wu.ac.th](mailto:saruda.ku@wu.ac.th)

Frederick Ramirez Masangkay: frederick_masangkay2002@yahoo.com

Aongart Mahittikorn: [aongart.mah@mahidol.ac.th](mailto:aongart.mah@mahidol.ac.th)

Kwuntida Uthaisar Kotepui: [kwuntida.ut@wu.ac.th](mailto:kwuntida.ut@wu.ac.th)

**Table S4. Meta-regression results**

| **Meta-analysis of UA** | **Covariates** | ***P* value** | **tau2** | **I^2^ (%)** | **R-squared (%)** | **Number of studies** |
| --- | --- | --- | --- | --- | --- | --- |
| **Malaria patients vs uninfected individuals** | Publication years | 0.04 | 1.17 | 95.38 | 4.86 | 16 |
|  | Study design | < 0.01 | 0.92 | 94.46 | 24.75 | 16 |
|  | Continent | 0.53 | 1.34 | 96.09 | 0 | 16 |
|  | Age group | 0.87 | 1.50 | 96.51 | 0 | 16 |
|  | *Plasmodium* species | 0.84 | 1.56 | 96.47 | 0 | 16 |
|  | Diagnostic method for malaria | 0.88 | 1.44 | 96.34 | 0 | 16 |
|  | Types of blood samples | 0.24 | 1.37 | 96.1 | 0 | 16 |
| **Severe vs non-severe malaria** | Publication years | 0.52 | 11.50 | 98.95 | 0 | 6 |
|  | Study design | 0.62 | 11.59 | 98.95 | 0 | 6 |
|  | Continent | 0.56 | 11.08 | 98.89 | 0 | 6 |
|  | Age group | 0.56 | 11.08 | 98.89 | 0 | 6 |
|  | *Plasmodium* species | N/A | N/A | N/A | N/A | 6 |
|  | Diagnostic method for malaria | 0.06 | 12.19 | 98.98 | 0 | 6 |
|  | Types of blood samples | N/A | N/A | N/A | N/A | 6 |

N/A, not assessed because of collinearity
