# Supplementary material for: Association of uric acid levels with severity of Plasmodium infections: a systematic review and meta-analysis
Source: Sci Rep. 2023 Sep 11;13:14979. doi: 10.1038/s41598-023-42217-8 (PMC10495360; doi:10.1038/s41598-023-42217-8)
Supplement: Supplementary file 5 — Supplementary Table 5. [file 41598_2023_42217_MOESM5_ESM.docx]

**Association of uric acid levels with severity of *Plasmodium* infections: A systematic review and meta-analysis**

Saruda Kuraeiad^1^, Kwuntida Uthaisar Kotepui^1^, Frederick Ramirez Masangkay^2^, Aongart Mahittikorn^3*^, Manas Kotepui^1^*

^1^Medical Technology, School of Allied Health Sciences, Walailak University, Tha Sala, Nakhon Si Thammarat, Thailand

^2^Department of Medical Technology, Faculty of Pharmacy, University of Santo Tomas, Manila, Philippines

^3^Department of Protozoology, Faculty of Tropical Medicine, Mahidol University, Bangkok, Thailand

**^*^Corresponding author**

Manas Kotepui: manas.ko@wu.ac.th

Saruda Kuraeiad: [saruda.ku@wu.ac.th](mailto:saruda.ku@wu.ac.th)

Frederick Ramirez Masangkay: frederick_masangkay2002@yahoo.com

Aongart Mahittikorn: [aongart.mah@mahidol.ac.th](mailto:aongart.mah@mahidol.ac.th)

Kwuntida Uthaisar Kotepui: [kwuntida.ut@wu.ac.th](mailto:kwuntida.ut@wu.ac.th)

**Table S5. Nonparametric trim-and-fill analysis of publication bias**

| Studies | Hedges's g (95% CI) |
| --- | --- |
| Observed | 0.993 (0.881–1.106 |
| Observed + Imputed | 0.993 (0.881–1.106 |

Abbreviation: CI, confidence interval

Details of analysis: Linear estimator, imputing on the left

Number of studies = 19

Model: Random-effects, observed = 19

Method: DerSimonian–Laird, imputed = 0

Pooling Model: Random-effects; Method: DerSimonian–Laird
